# Preservation of Gastrointestinal Mucosal Barrier Function and Microbiome in Patients With Controlled HIV Infection

**DOI:** 10.3389/fimmu.2021.688886

**Published:** 2021-05-31

**Authors:** Gerald Mak, John J. Zaunders, Michelle Bailey, Nabila Seddiki, Geraint Rogers, Lex Leong, Tri Giang Phan, Anthony D. Kelleher, Kersten K. Koelsch, Mark A. Boyd, Mark Danta

**Affiliations:** ^1^ St. Vincent’s Clinical School, UNSW, Darlinghurst, NSW, Australia; ^2^ Centre for Applied Medical Research, St Vincent’s Hospital, Sydney, NSW, Australia; ^3^ Kirby Institute, UNSW Sydney, Sydney, NSW, Australia; ^4^ IDMIT Department/IBFJ, Immunology of Viral Infections and Autoimmune Diseases (IMVA), INSERM U1184, CEA, Université Paris Sud, Paris, France; ^5^ South Australian Health and Medical Research Institute (SAHMRI), Adelaide, SA, Australia; ^6^ Faculty of Science, Flinders University, Adelaide, SA, Australia; ^7^ Microbiology and Infectious Diseases, South Australia (SA) Pathology, Adelaide, SA, Australia; ^8^ Immunology Division Garvan Institute of Medical Research, Sydney, NSW, Australia; ^9^ Faculty of Health and Medical Sciences, University of Adelaide, Adelaide, SA, Australia; ^10^ Department of Gastroenterology, St. Vincent’s Hospital, Sydney, NSW, Australia

**Keywords:** HIV, CD4, antiretroviral therapy (ART), gut-associated lymphoid tissues (GALT), microbiome

## Abstract

**Background:**

Despite successful ART in people living with HIV infection (PLHIV) they experience increased morbidity and mortality compared with HIV-negative controls. A dominant paradigm is that gut-associated lymphatic tissue (GALT) destruction at the time of primary HIV infection leads to loss of gut integrity, pathological microbial translocation across the compromised gastrointestinal barrier and, consequently, systemic inflammation. We aimed to identify and measure specific changes in the gastrointestinal barrier that might allow bacterial translocation, and their persistence despite initiation of antiretroviral therapy (ART).

**Method:**

We conducted a cross-sectional study of the gastrointestinal (GIT) barrier in PLHIV and HIV-uninfected controls (HUC). The GIT barrier was assessed as follows: *in vivo* mucosal imaging using confocal endomicroscopy (CEM); the immunophenotype of GIT and circulating lymphocytes; the gut microbiome; and plasma inflammation markers Tumour Necrosis Factor-α (TNF-α) and Interleukin-6 (IL-6); and the microbial translocation marker sCD14.

**Results:**

A cohort of PLHIV who initiated ART early, during primary HIV infection (PHI), n=5), and late (chronic HIV infection (CHI), n=7) infection were evaluated for the differential effects of the stage of ART initiation on the GIT barrier compared with HUC (n=6). We observed a significant decrease in the CD4 T-cell count of CHI patients in the left colon (p=0.03) and a trend to a decrease in the terminal ileum (p=0.13). We did not find evidence of increased epithelial permeability by CEM. No significant differences were found in microbial translocation or inflammatory markers in plasma. In gut biopsies, CD8 T-cells, including resident intraepithelial CD103+ cells, did not show any significant elevation of activation in PLHIV, compared to HUC. The majority of residual circulating activated CD38+HLA-DR+ CD8 T-cells did not exhibit gut-homing integrins α4ß7, suggesting that they did not originate in GALT. A significant reduction in the evenness of species distribution in the microbiome of CHI subjects (p=0.016) was observed, with significantly higher relative abundance of the genus *Spirochaeta* in PHI subjects (p=0.042).

**Conclusion:**

These data suggest that substantial, non-specific increases in epithelial permeability may not be the most important mechanism of HIV-associated immune activation in well-controlled HIV-positive patients on antiretroviral therapy. Changes in gut microbiota warrant further study.

## Introduction

Chronic HIV-1 infection is associated with persistent elevated systemic immune activation, including increases in levels of pro-inflammatory cytokines (1), lymph node germinal centre activity, immunoglobulin secretion by B-cells (2, 3) and activation and increased turnover of T-cells (4), particularly including target CCR5+ CD4 T-cells (5). A proposed cause for this is gut microbial translocation, which is the pathological translocation of luminal micro-organisms from the gastrointestinal tract (GIT) to the portal and systemic circulation as a consequence of depletion and impaired reconstitution of gut-associated lymphoid tissue (GALT) CD4 T-cells (6, 7).

Effects of HIV in the GIT include epithelial apoptosis (8, 9) and loss of epithelial barrier integrity (10, 11) with evidence of increased epithelial tight junction permeability (12, 13). Focal loss of CD4 T-cells in the mucosa (14–16) and dysregulation of T-cell subtypes (17–20) have also been implicated. Microbial translocation is believed to lead to systemic immune activation, seen as a correlation between plasma LPS levels and circulating activated CD38+HLA-DR+ CD8 T-cells (7). Furthermore, gut microbiome composition correlates with increased immune activation in HIV-infected individuals (21–23). The effect of antiretroviral therapy (ART) on the gut microbiome and mucosal and systemic lymphocytes suggests partial but not complete normalization of the dysbiosis resulting from HIV-1 infection (24). Therefore, there is a need to further study the relationship between enduring changes in the microbiome, the mucosal barrier and systemic immune responses during ART.

Studies to date directly assessing the functional integrity of the intestinal barrier in HIV-infected individuals have generally investigated impairment using immunohistochemical or transcriptional analysis of biopsies (8, 11–13). Conventional techniques such as mannitol and lactulose permeability measuring intestinal barrier function (25) have also been used to study HIV-1 infected subjects (8, 9).

Confocal endomicroscopy (CEM) shows promise for accurate, focal analysis of the intestinal barrier *in vivo*. CEM is a novel technique utilising a laser confocal endomicroscope integrated into a colonoscope, gathering images at 1000x magnification (26, 27). CEM has been successful in identifying gastrointestinal barrier changes in inflammatory bowel disease (28, 29), in which microbial translocation is thought to play a role (30). To the best of our knowledge, this technique has only been used once to study the gastrointestinal barrier of PLHIV. This was done in a set of mainly elite controllers, with evidence of permeability, but no quantitative comparison to HIV-uninfected controls (31).

Our study aimed to confirm and quantify the increased permeability of the gut mucosal barrier, as directly observed *in vivo* using CEM, compared to HIV-uninfected controls, and explore potential relationships between gut microbiome, mucosal immune function, intestinal barrier integrity and markers of immune activation in PLHIV who commenced ART during either primary or established chronic HIV infection. All parameters were compared to HIV-uninfected controls.

## Methods

### Subjects

This cross-sectional pilot cohort study enrolled PLHIV who initiated ART during primary (PHI) and chronic (CHI) infection and had maintained virological control for >2 years, and a control group of HIV-uninfected controls (HUC). The HUC were matched for age and sex. PLHIV were considered treated in primary HIV infection (PHI) if ART was initiated within six months of HIV infection (HIV), and in chronic HIV infection (CHI) if treatment was initiated at least 12-months after HIV infection, as defined in the PINT study (32). Volunteers were excluded if they were unfit for colonoscopy, had a fluorescein allergy, or had specific inflammatory gastrointestinal conditions associated with colitis. This study was approved by the St Vincent’s Human Research Ethics Committee (HREC 14/214). All participants provided written informed consent.

### Confocal Endomicroscopy (CEM)

Colonoscopy with confocal endomicroscopy was conducted by a single colonoscopist using an Optiscan CIS-2 prototype confocal laser endomicroscope (Notting Hill, VIC, Australia). This device replaces one of two air/water channels of a conventional Olympus CF-H180AL endoscope (Tokyo, Japan) with a 488nm laser microscope. The endoscopic probe was applied perpendicular to the mucosal surface, and serial microscopic images of the terminal ileum were captured at scanning depths of 15-70µm during and after intravenous administration of 5mL 10% fluorescein sodium contrast (Alcon, Australia) in 1mL increments.

**Figure 1:**
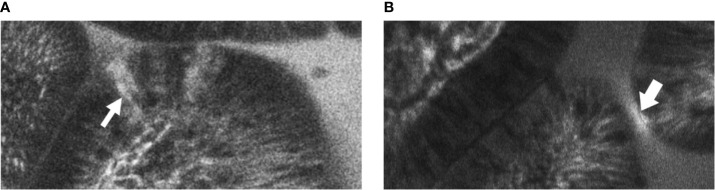
Example images of terminal ileal villi exhibiting **(A)** fluorescein leak and **(B)** cell junction enhancement **(A)** Cell junction enhancement, characterised by an area of increased fluorescence from the basal to the apical surface between two epithelial cells. **(B)** Fluorescein leak, characterised by increased fluorescence in the epithelial cell layer, and a distinct plume of contrast leakage into the lumen.

Two blinded observers reviewed the images with patient grouping identifiers removed. Images where villi and lumen were indistinguishable were discarded. The two observers then independently analysed the remaining images for evidence of cell junction enhancement and fluorescein leak (see **Figure 1**). Cell junction enhancement was defined as an area of increased, equal fluorescence between two epithelial cells, extending from the basal to the apical surface of the cell layer. Fluorescein leak was characterised by a distinct plume of contrast leakage into the lumen stemming from the epithelial luminal border. Both features have previously been used in studies to gauge mucosal barrier function and permeability in gastrointestinal pathologies (28, 29, 33, 34).

### Flow Cytometry

Ten pinch biopsies each were taken from the terminal ileum and left colon using endoscopic biopsy forceps during colonoscopy and separately stored in containers of media solution containing 10mL Roswell Park Memorial Institute (RPMI) culture media (Invitrogen, USA) with 10% foetal bovine serum (Bovogen, Australia) and 100U PenStrep (Invitrogen). Blood was concurrently collected in Vacutainer tubes with sodium heparin anti-coagulant (Becton Dickinson, NJ, USA).

Biopsy samples were weighed, then minced with sterile scissors, before enzymatic digestion using collagenase type III (Sigma-Aldrich) and DNase (Sigma-Aldrich), in order to prepare single cell suspensions for flow cytometry, as previously described (35). All samples were stained for surface markers according to manufacturer instructions, as previously described (35). Additionally, samples from the terminal ileum and left colon were stained for epithelial cells using the monoclonal antibody EpCam-FITC (BD Biosciences, CA, USA). Samples were washed with PBS (Dulbecco’s Phosphate Buffered Saline (DPBS) with 0.5% BSA and 0.1% sodium azide) and fixed in a solution of 0.5% paraformaldehyde in DPBS. Peripheral blood samples were stained, lysed and fixed as previously described (35).

Cells were analysed using a four-laser LSR-II flow cytometer (BD Biosciences) and BD FACSDiva version 8.0 (BD Biosciences), then further analysed using FlowJo version 10.7.1 (Ashland, OR). The gating strategy for lymphocytes isolated from gastrointestinal biopsies has been previously described (35), and is shown in **Supplementary Figure 1A**. Gating of peripheral blood lymphocytes utilised a similar strategy as shown in **Supplementary Figure 1B**.

### Microbiome Analysis

Stool samples were collected and preserved using OMNIgene GUT microbial stabilisation kit (DNA Genotek, Ontario, Canada). Genomic DNA was extracted using DNeasy PowerLyzer PowerSoil DNA isolation kits (Qiagen, Hilden, Germany) as per manufacturer’s instructions. 16S rRNA sequencing was performed by first preparing the amplicon library using a previously described protocol (36), and sequenced on the Illumina Miseq sequencing platform using Illumina Miseq v3 kit with 2 x 300 bp cycle (Illumina Inc., CA, USA). Downstream processing of the amplicon sequencing reads was carried out as described (36). Briefly, reads were quality filtered and merged, followed by assigning operational taxonomic units (OTUs) using the Quantitative Insights into Microbial Ecology (37) software. No samples were eliminated following subsampling to the depth of 8,079 reads.

### Plasma Marker Analysis

The plasma level of soluble CD14 (sCD14) was used as a marker of monocyte activation by lipopolysaccharide and microbial translocation. Other cytokines included TNF and IL6. EDTA anti-coagulated blood was centrifuged at 1600rpm for 15 minutes to obtain plasma for testing in ELISAs using commercially available kits for sCD14, IL-6 and TNF-α (all R&D systems, MN, USA) according to manufacturer’s instructions.

### Data Analysis

CD4 and CD8 T-cells in gastrointestinal biopsies at each sample site were counted as absolute cell numbers as previously described (35), but were also normalised by weight of biopsies and by the number of epithelial cells in the biopsies (35). Data was analysed using Prism software version 9.0 (GraphPad, La Jolla, CA). Quantitative analysis was performed using Kruskal-Wallis one-way ANOVA, with two-tailed Mann-Whitney U post-hoc analysis. Spearman’s correlation was used to compare two continuous variables. Cohen’s kappa score was used to measure inter-observer agreement in confocal endomicroscopy image interpretation. Faecal microbiota variation was analysed using Shannon and Simpson diversity indices, while group diversity as calculated using Bray-Curtis dissimilarity distance was illustrated using non-metric multidimensional scaling (NMDS) ordination, and tested using permutational multivariate analysis of variance (PERMANOVA). Kruskal-Wallis with Benjamini-Hochberg false-discovery rate adjustment was employed to assess significant difference in specific bacterial taxa between groups.

## Results

### Participants

**Figure 2:**
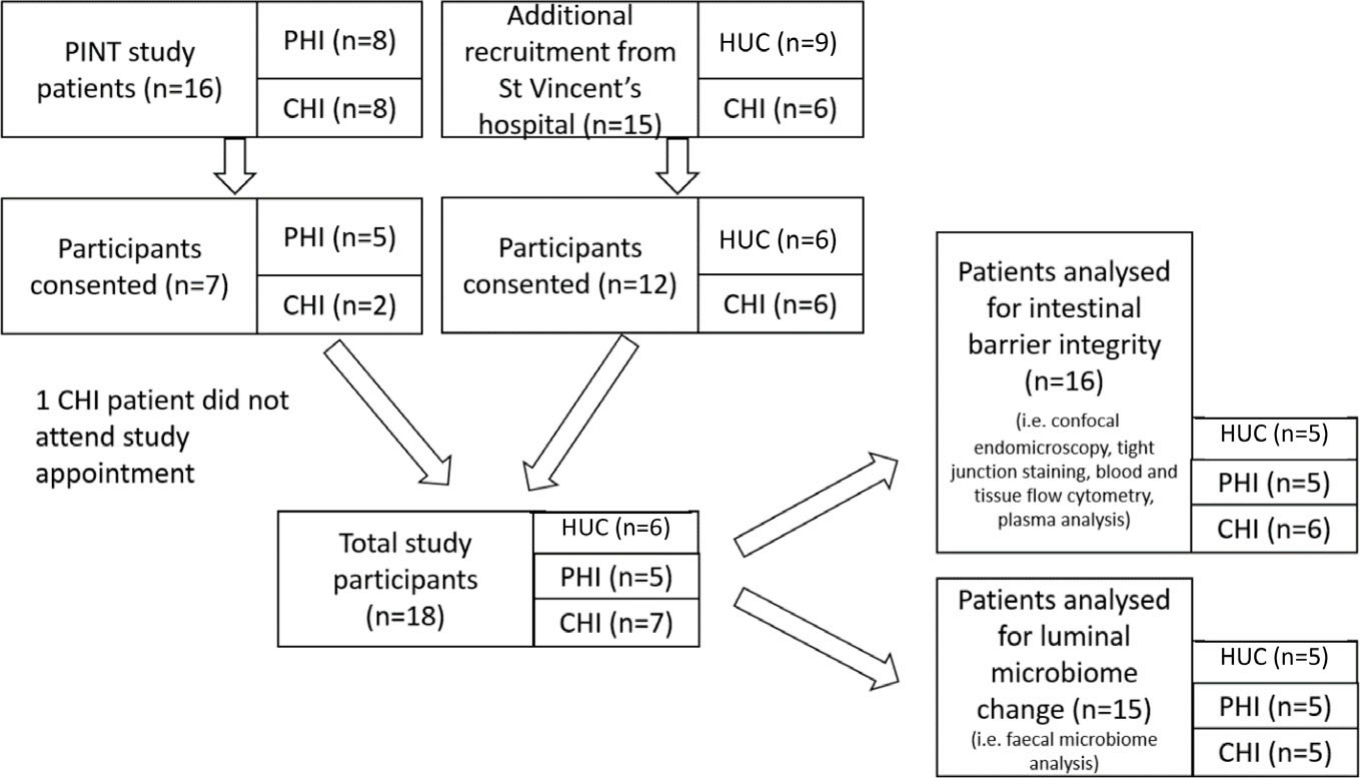
Patient recruitment flowchart outlining selection of patients for confocal endomicroscopy imaging, tight junction, blood and tissue lymphocyte, plasma marker and faecal microbiome analyses. HUC, HIV-uninfected control; PHI, HIV-positive participant who initiated antiretroviral therapy during primary HIV infection; CHI, HIV-positive participant who initiated antiretroviral therapy during chronic HIV infection.

Of the 16 HIV-positive participants from the PINT study, which prospectively studied the effect of commencing a raltegravir-containing regimen during either primary HIV-1 infection (PHI) or chronic infection (CHI) (32), five primary and two chronic HIV participants re-enrolled into this study. A further six HIV-positive participants and six HIV-uninfected controls (HUC) were recruited from outpatient clinics at St Vincent’s Hospital, Sydney. One patient in the PHI group did not attend for study procedures. Hence, a total of six HUC, five PHI and seven CHI subjects attended their allocated study session (**Figure 2**). All participants were male, with baseline characteristics outlined in **Table 1**. The CHI subjects had ART for a median 7 years (range 4-23) and the PHI subjects for 7 years (range 7-7).

**Table 1:** Baseline characteristics and clinical parameters of study participants.

	HIV-Uninfected controls (n=6)	HIV-positive participants treated with antiretroviral therapy in primary infection (n=5)	HIV-positive participants treated with antiretroviral therapy in chronic infection (n=7)	p-value
**Age** years median (range)	49 (33-58)	52 (48-55)	49 (35-53)	0.42
**Gender** Male n (%)	6 (100%)	5 (100%)	7 (100%)	1.00
**Years since diagnosis of HIV** median (range)	–	7 (7-7)	17.5 (8-33)	<0.01
**Years since commencement of ART** median (range)	–	7 (7-7)	6.5 (4-23)	0.48
**Time between diagnosis and commencement of ART** years median (range)	–	0 (0-0)	7 (2-20)	<0.01
**Current antiretroviral therapy regimen**	–	RAL/TDF/FTC	TDF/FTC/NVP	–
		RAL/TDF/FTC	EVG/c/TDF/FTC	
		RAL/TDF/FTC	EVG/c/TDF/FTC	
		EVG/c/TDF/FTC	RPV/TDF/FTC	
		DTG/TDF/FTC	RAL/TDF/FTC	
			ABC/3TC	
			ABC/3TC/NVP/RAL	
**CD4 T-cell count on visit** cells/mm^3^ median (range)*	881.9 (748.3-1278)	792.2 (421.5-888.3)	708.9 (602.1-809.8)	0.11
**CD8 T-cell count on visit** cells/mm^3^ median (range)*	734.8 (658.8-1045)	519.8 (264.5-1305)	733.6 (523.7-2417)	0.65
**Last measured plasma viral load**	–	Undetectable	Undetectable	–
**Acute infections or vaccinations in past month n** (%)	3 (50%)	0 (0%)	3 (43%)	0.06
**Antibiotic use in past month** Yes n (%)	1 (17%)	0 (0%)	2 (29%)	0.42
**Overseas travel in past year** Yes n (%)	3 (50%)	1 (20%)	4 (57%)	0.42
**Current Smoker** Yes n (%)	0 (0%)	1 (20%)	2 (29%)	0.38
**Current Alcohol Use** Yes n (%)	5 (83%)	4 (80%)	6 (86%)	0.97

*Systemic lymphocyte counts were only analysed in patients where confocal imaging, blood and tissue samples were collected. Chi-square test was used to analyse categorical data for significance. Mann Whitney U and Kruskal Wallis tests were used to analyse continuous data for 2 and >2 groups, respectively. RAL, raltegravir; TDF, tenofovir disoproxil fumarate; FTC, emtricitabine; EVG, elvitegravir; DTG, dolutegravir; NVP, nevirapine; RPV, rilpivirine; ABC, abacavir; 3TC, lamivudine.

One patient from the CHI group and one patient from the HUC group were excluded from confocal endomicroscopy and lymphocyte analysis due to inadequate visualisation of the terminal ileum, confocal imaging calibration issues, or non-attendance. One patient in the HUC group and two patients in the CHI group were excluded from microbiome analysis due to inadequate faecal sample.

### Preservation of Gastrointestinal Mucosal Barrier Integrity Using Confocal Endomicroscopy (CEM)

The median time taken to examine and capture the CEM images in the terminal ileum per patient was 9 minutes (range 6-20). A median of 889 (382-2046) images taken per patient were analysed after removal of 282 (22-993) unfocused images per patient. Fluorescein leak (**Figure 1A**) and cell junction enhancement (**Figure 1B**) were able to be identified by the two observers. Of images identified by one or the other observer to contain fluorescein leak or cell junction enhancement, 29.8% were identified by the other observer. Inter-observer agreement for the presence of features identified per patient was moderate (κ=0.43) for cell junction enhancement and substantial (κ=0.75) for fluorescein leak. There was also a strong correlation between the total number of features identified by each observer separately and features identified by both observers (Spearman r 0.81; 95%CI 0.51-0.93; p<0.01).

**Figure 3:**
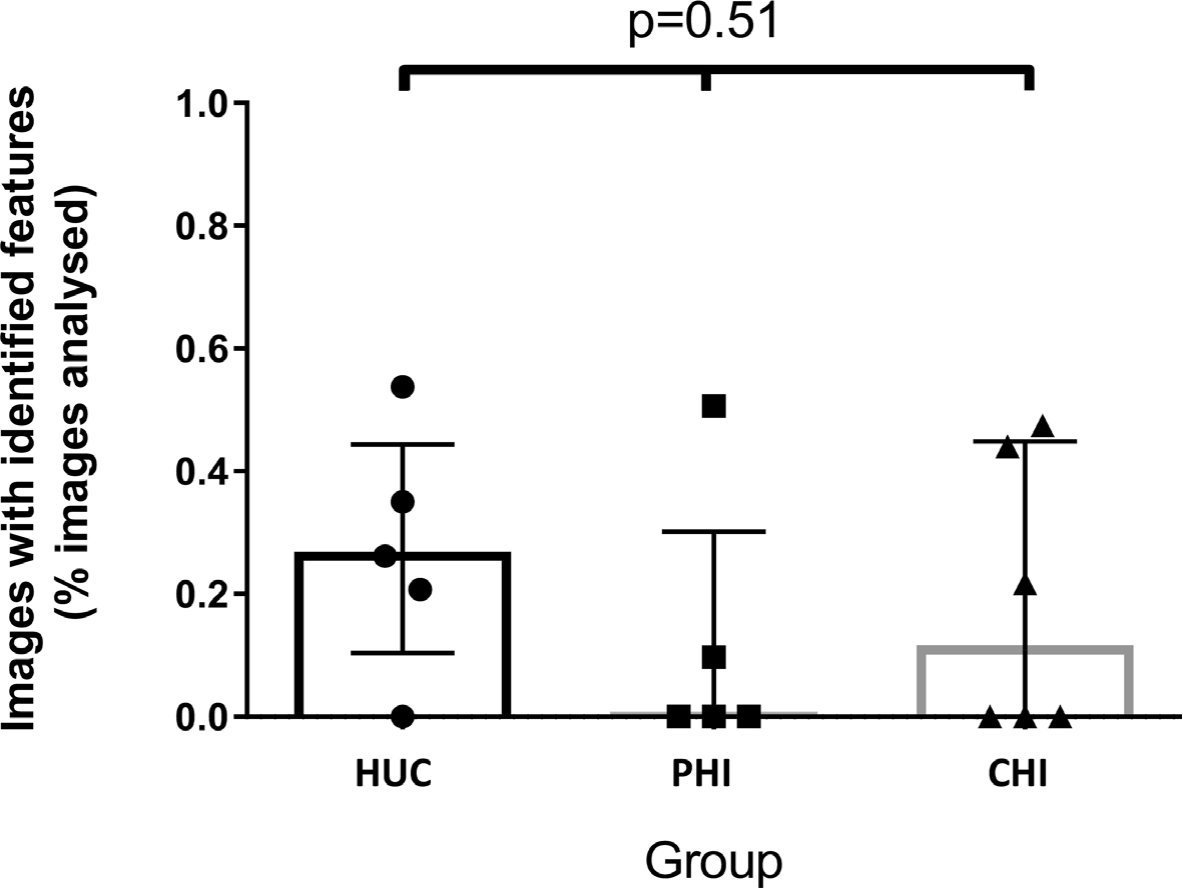
Images with identified confocal endomicroscopy features (fluorescein leak and cell junction enhancement) as a percentage of total images analysed for the participant, across three groups. Median percentage of images was 0.26% for HIV-uninfected controls (HUC), 0.00% for HIV-positive participants treated in primary infection (PHI), and 0.11% for HIV-positive participants treated in chronic infection (CHI). Statistical analysis was conducted using Kruskal Wallis one-way analysis of variation.

The number of images with fluorescein leak and cell junction enhancement was small as a proportion of the total number of images (median 0.15%, range 0-0.54%). There was no statistically significant difference in the median percentage of CEM features seen in participants across the three groups (HUC=0.26% *vs* PHI 0% *vs* CHI 0.11%; p=0.51) (**Figure 3**). No significant differences were found when fluorescein leak and cell junction enhancement were analysed separately (data not shown).

### Characterisation of CD4 and CD8 T-Cells in Gastrointestinal Tissue

In addition to absolute counts of CD4 and CD8 T-cells in gastrointestinal biopsies at each sample site, as previously described (35), numbers of these cells were also normalised by weight of biopsies and also by the number of epithelial cells in the biopsies (35). This revealed similar weights and epithelial cell counts between groups, but no correlation between the weight and epithelial cell count.

**Figure 4:**
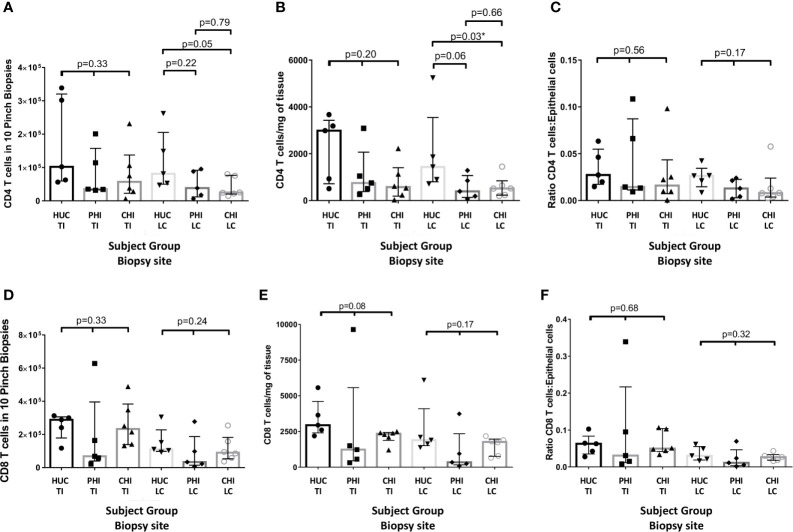
Changes in CD4 panels **(A–C)** and CD8 **(D–F)** cell numbers in the terminal ileum and left colon between patient groups as: **(A, D)** an absolute count from ten biopsies; **(B, E)** standardised per milligram of tissue; and **(C, F)** standardized by epithelial cell number HUC, HIV-uninfected controls; PHI, HIV-positive patients treated during primary infection; CHI, HIV-positive patients treated during chronic infection; TI, terminal ileum; LC, left colon, *statistically significant (p ≤ 0.05). Statistical analysis was conducted using Kruskal Wallis one-way analysis of variance with pairwise post-hoc testing using Mann Whitney U.

By absolute count from biopsies, there was no significant difference between CD4 T-cell counts in terminal ileum (TI) biopsies across the three study groups by Kruskal-Wallis test, but there was a significant difference between HUC and CHI groups in left colon (LC) biopsies (**Figure 4A**). By weight, there was an absolute decrease in CD4 T-cell numbers per mg in PLHIV in the LC compared with HUC participants, (HUC 1434.5 vs PHI 392.56 *vs* CHI 515.6 cells/mg; p=0.03) (**Figure 4B**). A similar decreasing trend was found in the TI samples (HUC 2987.8 *vs* PHI 751.8 *vs* CHI 577.9 cells/mg; p=0.20) (**Figure 4B**). There was no significant change in the CD4 T-cell count:epithelial cell number ratio in the TI (HUC 0.027 *vs* PHI 0.014 *vs* CHI 0.016; p=0.56) or LC (HUC 0.026 *vs* PHI 0.013 vs CHI 0.008; p=0.17) (**Figure 4C**).

There were no significant changes in the CD8 T-cell count by biopsy, weight or epithelial cell ratio, in either the TI or LC across the three groups (**Figures 4D–F**).

### Association Between Circulating and Gastrointestinal Lymphocyte Numbers

When analysing the samples of all volunteers in our study, a significant positive correlation was found between the CD4 T-cell count in peripheral blood and the number of CD4 T-cell count in gastrointestinal tissue in both the TI (p<0.01 r=0.76) and LC (p=0.02 r=0.57) (**Table 2**); the degree of correlation was higher in the TI than in the LC. A significant positive correlation was also found between peripheral blood CD8 T-cell count and CD8 T-cell number in the TI (p<0.01 r=0.69) (**Table 3**).

**Table 2:** Relationship between systemic CD4 T-cell concentration measured using peripheral blood and measured gastrointestinal lymphocyte numbers, for all study groups combined, and for HIV-positive study participants only.

	All Patients			HIV-positive Patients		
	n	p-value	r	n	p-value	r
CD4 T-cell count for all biopsies						
TI	16	<0.01**	0.76	11	0.03*	0.65
LC	16	0.02*	0.57	11	0.27	0.36
CD4 T-cell count per milligram of tissue						
TI	16	<0.01**	0.81	11	<0.01**	0.76
LC	16	0.02*	0.59	11	0.20	0.42
Ratio of CD4 T-cells:Epithelial cells						
TI	16	0.01*	0.61	11	0.19	0.43
LC	16	0.09	0.44	11	0.17	0.45

Spearman Correlation was used for analysis of significance. *p ≤ 0.05. **p ≤ 0.01.

**Table 3:** Relationship between systemic CD8 T-cell concentration measured in peripheral blood and measured gastrointestinal lymphocyte numbers, for all study groups combined, and for HIV-positive study participants only.

	All Patients			HIV-positive Patients		
	n	p-value	r	n	p-value	r
CD8 T-cell count for all biopsies						
TI	16	<0.01**	0.69	11	<0.01**	0.76
LC	16	0.07	0.46	11	0.03*	0.65
CD8 T-cell count per milligram of tissue						
TI	16	<0.01**	0.65	11	<0.01**	0.79
LC	16	0.17	0.36	11	0.08	0.56
Ratio of CD8 T-cells:Epithelial cells						
TI	16	<0.01**	0.67	11	<0.01**	0.80
LC	16	0.08	0.45	11	0.03*	0.68

Spearman Correlation was used for analysis of significance. *p ≤ 0.05. **p ≤ 0.01.

### CD8 T Cell Activation in Peripheral Blood and Biopsies

**Figure 5:**
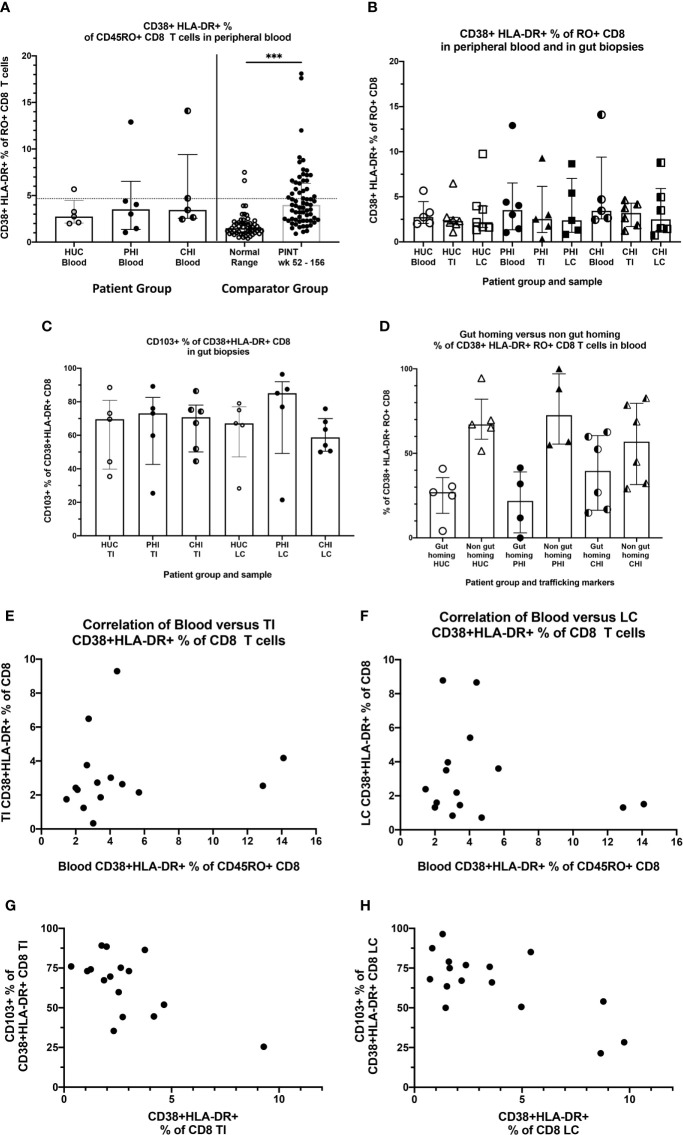
Activated CD38+HLA-DR+ CD8 T-cells in blood and tissues. **(A)** Activated CD38+HLA-DR+ as % of CD45RA- CD8 T cells in peripheral blood, by study group. HUC, HIV-uninfected controls; PHI, HIV-positive patients treated during primary infection; CHI, HIV-positive patients treated during chronic infection. The Comparator Groups are (left) normal range for HUC subjects from reference (38) and the 95^th^ percentile (4.6%) is shown as the dotted horizontal line, and (right) PINT subjects longitudinal observations from reference (38). Statistical analysis between HUC, PHI and CHI groups was conducted using Kruskal Wallis one-way analysis of variance. Statistical analysis between comparator normal range and PINT groups was done using Mann-Whitney test. **(B)** Comparison of activated CD38+HLA-DR+ as % of CD45RA- CD8 T-cells in peripheral blood, TI biopsies and LC biopsies, respectively by study group. **(C)** Percentage of activated CD38+HLA-DR+ CD8 T-cells in TI and LC biopsies that are also CD103+. **(D)** Percentage of activated CD38+HLA-DR+ CD8 T-cells in peripheral blood that are either CD49d+integrin ß7+ gut-homing or CD49d+integrin ß7+-negative non-gut-homing, by study group. **(E)** Correlation of activated CD38+HLA-DR+ as % of CD8 T-cells in peripheral blood with activated CD38+HLA-DR+ as % of CD8 T cells in TI biopsies. **(F)** Correlation of activated CD38+HLA-DR+ as % of CD8 T-cells in peripheral blood with activated CD38+HLA-DR+ as % of CD8 T cells in LC biopsies. **(G)** Correlation of CD103+ as % of activated CD38+HLA-DR+ CD8 T-cells in TI biopsies with activated CD38+HLA-DR+ as % of CD8 T cells in TI biopsies. **(H)** Correlation of CD103+ as % of activated CD38+HLA-DR+ CD8 T-cells in LC biopsies with activated CD38+HLA-DR+ as % of CD8 T cells in LC biopsies. ***p < 0.0001.

Circulating activated CD38+HLA-DR+ CD8 T-cells, as a proportion of total CD8 T-cells in the peripheral blood, was measured as a marker of systemic immune activation. No statistically significant differences were found across the percentages in the three subject groups of the current study (medians: HUC 2.75% PHI 3.53% CHI 3.45% Kruskal-Wallis test p=0.69) (**Figure 5A** left). As comparator data, a previous larger sample of HUC and all longitudinal data from the PINT study (38) showed that there was a small but significant elevation of activated CD38+HLA-DR+ CD8 T-cells in PLHIV on long-term suppressive ART (medians: HUC 1.5%, HIV+ PINT subjects 4.0%; Mann-Whitney test p<0.0001) (38) (**Figure 5A**, right). For the PLHIV in PINT, 25/67 observations were above 4.6%, which was the 95th percentile of the normal range.

We also measured the activated CD38+HLA-DR+ % of CD8 T-cells in the gut biopsies and compared them to the corresponding peripheral blood levels (**Figure 5B**). In general, the level of activation was in the same range as for circulating CD8 T-cells, and no significant elevations were seen in gut biopsies from PLHIV, compared to the HUC gut biopsies (**Figure 5B**).

When we further subdivided the activated CD38+HLA-DR+ CD8 T-cells in the gut biopsies into CD103+ resident intraepithelial cells (39) versus CD103- presumptively migratory cells (**Supplementary Figure 1A**), we found that, in most biopsies, the majority of CD38+HLA-DR+ CD8 T-cells were CD103+ tissue resident cells that are unlikely to recirculate (**Figure 5C**).

Conversely, we examined the expression of integrins α4ß7, that determine whether the cells traffic through GALT, on CD38+HLA-DR+ CD8 T-cells in peripheral blood (**Supplementary Figure 1B**). The results show that the majority of circulating activated CD38+HLA-DR+ CD8 T-cells do not express integrins α4ß7 (**Figure 5D**). We also did not see any correlation between the levels of circulating activated CD38+HLA-DR+ CD8 T-cells with the corresponding cells in either TI biopsies (**Figure 5E**) or LC biopsies (**Figure 5F**). Finally, when there were larger percentages of activated CD38+HLA-DR+ CD8 T-cells in gut biopsies, they were mostly CD103- in TI (**Figure 5G**) and in LC (**Figure 5H**), suggesting that they were migratory.

Overall, the results indicate that while it appears that there are slightly more circulating activated CD38+HLA-DR+ CD8 T-cells in PLHIV on fully suppressive ART, they appear to be more systemic in origin and not directly associated with activation within GALT.

We also used an alternative CD38+CD127- phenotype of activated CD8 T cells since it has been reported that levels of this phenotype differed between HIV+ subjects and uninfected controls, in both rectal mucosa and blood (40). When we studied these cells (**Supplementary Figure 1B**) we did not find any significant differences in CD38+CD127- CD8 T-cells between PLHIV and HIV-uninfected subjects in either blood or gut biopsies (**Supplementary Figure 2**).

### Plasma Markers of Immune Activation

**Figure 6:**
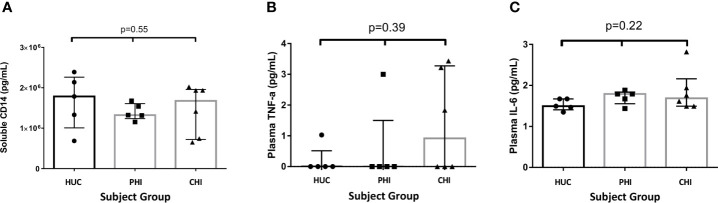
Levels of soluble proteins measured in plasma between the study groups. **(A)** Levels of soluble CD14 measured in plasma between the study groups. **(B)** Levels of TNF-α measured in plasma between the study groups. **(C)** Levels of IL-6 measured in plasma between the study groups. Statistical significance was calculated using the Kruskal Wallis test.

There were no significant differences between the levels of sCD14 (pg/mL) found in the three study groups (HUC 1.78x10^6^ *vs* PHI 1.32x10^6^ *vs* CHI 1.68x10^6^ pg/ml; p=0.55) (**Figure 6A**). The majority of study participants (68.75%) had undetectable concentrations of TNF-α in plasma (**Figure 6B**). A higher number of patients in the CHI group had detectable plasma TNF-α levels compared with those in both the HIV-uninfected and PHI groups (n=3, n=1, n=1 respectively), but this was not statistically significant. Of the five participants with detectable TNF-α concentrations, 4 (80%) had detectable cell junction enhancement or fluorescein leak seen on confocal endomicroscopy (HUC n=1, PHI n=1, CHI n=2). There were no significant differences in IL-6 concentrations across the three groups (HUC 1.49pg/mL *vs* PHI 1.79pg/mL *vs* CHI 1.69pg/mL; p=0.22) (**Figure 6C**). Finally, the relationship between epithelial integrity and both local immune parameters in the TI as well as systemic parameters was explored. No significant differences were found between the presence of confocal endomicroscopy features and the immune parameters measured (**Table 4**).

**Table 4:** Relationship of confocal endomicroscopy features identified in patients and measured parameters.

	All patient groups (n=16)			HIV-positive patients only (n=11)		
Parameter	CEM no abnormalities (n=7)	CEM abnormalities (n=9)	P	CEM no abnormalities (n=6)	CEM abnormalities (n=5)	P
Systemic CD4 (cells/mm^3^)	697.3	769.7	0.30	694.5	752.0	0.33
Systemic CD8 (cells/mm^3^)	769.0	674.3	0.84	733.6	661.6	0.79
Systemic activated CD8%	3.17	1.63	0.28	3.21	1.55	0.25
Terminal Ileum CD4 T-cells/mg	614.7	1133	0.41	577.9	1133	0.33
Terminal Ileum CD8 T-cells/mg	2105	2412	0.41	1803	2290	0.93
sCD14 (pgx10^6^/mL)	1.538	1.417	0.68	1.430	1.417	0.93
TNF-α (pg/mL)	0.000	0.000	1.00	0.000	3.003	0.11
IL-6 (pg/mL)	1.762	1.613	0.26	1.777	1.613	0.42

Median values are shown for each group. TI, terminal ileum; IgA, immunoglobulin A; sCD14, soluble CD14; TNF-α, tumour necrosis factor alpha; IL-6, interleukin 6. Analysis was performed using Mann Whitney U rank sum tests.

### Characterisation of Gut Microbiota

**Figure 7:**
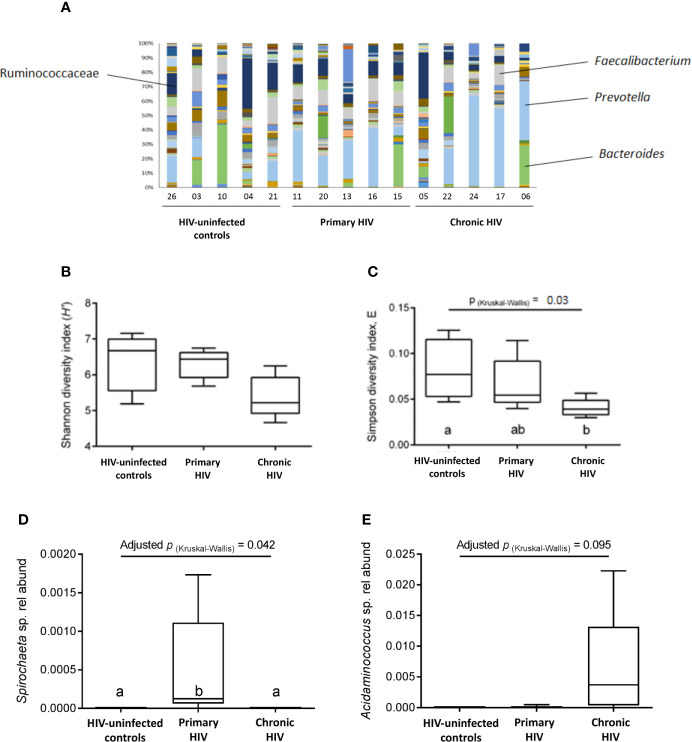
Faecal microbiota composition. **(A)** Bacterial composition from analysis of faecal samples from study participants prior to bowel preparation. A total of 145 types of taxa are represented, with increased representation of fermentative taxa (i.e., Ruminococcaceae, Faecalibacterium, Prevotella, Bacteroides) compared to other taxa. **(B)** Species diversity and taxa distribution in study participants as measured by Shannon diversity index (H’) **(C)** Species diversity and taxa distribution in study participants as measured by Simpson diversity index E **(D)** Relative abundance of the *Spirochaeta* genus between groups. **(E)** Relative abundance of the *Acidaminococcus* genus between groups.

Microbiota composition across all patients was dominated by fermentative bacterial taxa, including members of the *Faecalibacterium* (median 0.10; interquartile range [IQR] 0.05-0.12), *Prevotella* (median 0.21; IQR 0.09-0.38) and Bacteroides (median 0.01, IQR 0.003-0.12) genera (**Figure 7A**). There was no significant difference between groups in overall microbiota composition based on Bray Curtis dissimilarity, as assessed by PERMANOVA (P(perm)=0.202, pseudo-F=1.272, 9551 permutations). A non-significant decreasing trend in taxa diversity was also found using the Shannon diversity index H’ (**Figure 7B**). However, a significant difference (p=0.03) was found between PLHIV and HIV-uninfected participants in the distribution evenness of taxa, as measured by the Simpson diversity index (**Figure 7C**). The relative abundance of the *Spirochaeta* genus was significantly higher in the PHI group (p=0.042; **Figure 7D**), while the *Acidaminococcus* genus showed a non-significant trend towards increased relative abundance in the CHI group (**Figure 7E**). Patients in the CHI group exhibited a trend of increasing *Prevotella* relative abundance, and a trend of decreasing *Faecalibacterium* relative abundance. There was no correlation between the microbiota and the CEM or the cytokines (data not shown).

## Discussion

In this study, we aimed to confirm and characterise the *in vivo* intestinal permeability of study participants with treated HIV infection, compared to HIV-uninfected controls, using CEM, as well as define associations with parameters of mucosal barrier integrity and systemic immune activation and inflammation. Contrary to expectations, however, we could not confirm significant differences for *in vivo* intestinal permeability between study groups.

In our study methodology, the amount of fluorescein used was matched with other studies which successfully identified changes in gastrointestinal villi, determining the timeframe in which contrast-enhanced images could be taken with the confocal microscope (28, 29). Fluorescein leak and cell junction enhancement were features chosen as indicative of increased epithelial permeability in intestinal villi as interpretation could be easily standardised and due to their previous association with damage to epithelial barrier integrity in other contexts (41). These features were identifiable in our images, but, importantly, there were no significant quantitative differences between the subject groups.

A recent study had also utilized *in vivo* microscopic imaging of rectal mucosa and reported that there was increased fluorescein leakage and intramucosal bacteria in most of the 10 HIV+ subjects studied (31). However, unlike our study, there was no quantitative comparison to HIV-uninfected controls, and 7/10 HIV+ subjects were Elite Controllers (31), who are not representative of PLHIV on long-term ART.

Although *in vitro* studies have reported increased tight junction permeability, epithelial inflammation and apoptosis (10) on direct exposure to the HIV-1, this effect could be reduced *in vivo* due to the fast turnover rate of epithelial cells (42) and sustained suppression of HIV replication with long term ART. Certainly, this would be consistent with both this small study’s results and results from an *in vitro* study of intestinal permeability by Epple et al. in 2009 (12). Although inter-observer agreement on cell junction enhancement was low compared to other studies (28, 41, 43), the comparable agreement on presence of fluorescein leak with other studies, and the high percentage of study participants with CEM features suggest the methodology is sufficiently sensitive. Larger studies utilising this method in treated and untreated PLHIV with comparison to other inflammatory gastrointestinal diseases, such as ulcerative colitis or Crohn’s disease, would help place these findings in a clearer context.

Ours is the first study to directly explore the effect of early and late initiation of effective ART on intestinal permeability. A secondary hypothesis was that gastrointestinal lymphocyte depletion would be more profound in PLHIV who commenced ART during CHI. Consistent with this hypothesis, our study found a significant decrease in LC CD4 T-cell counts and a decreasing trend in TI CD4 T-cell counts of CHI participants compared to HIV-uninfected controls, and is consistent with our previous studies (35). This result is supported in previous studies on T-lymphocyte cell counts in gastrointestinal tissue (14, 16, 44) in untreated and treated (6, 44–46) PLHIV, but the effect of early ART initiation in the literature is unclear. While studies have reported limited reconstitution of the proportion of CD4 T-cells in intestinal tissue even when treated during primary infection (6, 47), a study by Allers et al. on absolute CD4 T-cell numbers reported treatment during early HIV infection led to complete preservation of CD4 T-cells in duodenal tissue (45).

Our study found a decreasing trend in LC CD4 T-cell numbers of PLHIV treated in primary infection. The association was less clear in the TI. Data from a study by Yukl et al. (48) previously showed a larger quantifiable decrease in the proportion of CD4 T-cells in ileal samples of HIV-positive volunteers than in rectal samples. It has been suggested that the effect of HIV infection on T-cell numbers is variable depending on the gastrointestinal sample site due to different frequencies of gut-homing and regulatory T cells (48). A possible identified contributor to the variability of lymphocyte counts was the nature of lymphoid follicles being concentrated in Peyer’s patches in the TI (49), making representative sampling of this site difficult. Our group’s larger analysis of gastrointestinal lymphocyte counts have revealed significant decreases in CD4 T-cells in both the LC and TI, using both absolute cell counts and epithelial cell ratio, although in that study samples were not analysed by weight (35).

Earlier studies quantified the CD4 T-cells as a percentage of total CD3+ T-cells. However, absolute CD8+ CD3+ T-cell levels may be increased in HIV-positive patients with untreated infection, with high variability in the levels in patients on ART (14, 45), resulting in either a decrease in, or increased variability, in mucosal CD4 T-cell percentages. This study is one of few to use absolute lymphocyte counts to preclude the effects of CD8 T-cell proliferation. As a standard method of normalisation is not documented, both epithelial cell counts and biopsy weight were used as normalisation methods due to possible variations in size of biopsy samples. However, we found stronger correlations between peripheral blood and tissue lymphocytes when normalising by weight. This suggests that lymphocyte loss may be more concentrated in areas of lymphoid aggregation, which are deeper in the tissue, compared to tissue-resident lymphocytes near the epithelial border. Weight may be more representative of biopsy volume, and epithelial cells representative of total biopsy tissue area. This might be a useful area of investigation, which could involve comparison of immuno-histochemistry and flow cytometry methods.

Despite systemic CD4 activation being central to HIV pathogenesis (50), we have concentrated on the study of activated CD38+HLA-DR+ CD8 T-cells, rather than activation of CD4 T-cells for several reasons. Firstly, levels of activated CD38+HLA-DR+ CD8 T-cells in blood samples from untreated patients have been clearly correlated with disease progression (51). Secondly, in patients receiving ART, we and others have documented residual elevation of activated CD8 T-cells (38, 52, 53) despite suppression of HIV replication; these levels have been correlated with plasma LPS levels, consistent with the microbial translocation hypothesis (7). Finally, the frontline CD8 T-cells in GALT can be identified as CD103+ intraepithelial resident memory cells, and these cells should be most activated by barrier dysfunction. In contrast, levels of CD4 activation in gut biopsy samples are complicated by the presence of germinal centres containing activated CD4 T follicular helper cells (35), and these cells do not leave GALT.

Instead, we have found that there was no difference in CD8 T-cell activation in gut biopsy samples between PLHIV on ART compared to HIV-uninfected controls, and in particular, we documented for the first time that CD103+ CD8 T-cells were generally more activated than CD103- migratory CD8+ T-cells. Furthermore, there was only a minority of activated CD8 T-cells in peripheral blood that expressed the gut-homing integrins α4ß7, and there was no obvious correlation of levels of activation of CD8 T-cells between the GALT and blood compartments. Altogether, our detailed results suggest that it is unlikely that the main source of activated CD8 T-cells in blood is due to gastrointestinal barrier dysfunction.

We have previously found that crucial CD4 T follicular helper cells in GALT are not significantly depleted in HIV+ subjects on ART, nor are their important downstream effector cells, IgA+ B-cells (35), which will help maintain microbial homeostasis of this tissue. Instead, in these GALT germinal centres as well as in other lymphoid tissues, it is possible that CD4 T follicular helper cells act as a residual underlying HIV reservoir (3, 54) and may contribute to lingering CD8 T-cell activation. Undoubtedly, GALT CD4 T-cells continue to contribute to the HIV reservoir under ART (55, 56). However, we have previously found that in the circulation, gut-homing CD4 T cells only contain a small proportion of the HIV-1 DNA in PBMC (57). Other tissues, despite fully suppressed plasma viremia, are likely to contain HIV-1 reservoirs that activate CD8 T-cells, such as CD4 T follicular helper cells in peripheral lymph nodes (3, 54), infected alveolar macrophages (58) and infected cells in the CNS (59, 60).

An increased relative abundance of *Spirochaeta*, *Prevotella*, *Acidaminococcus* species in those with HIV, and a reduced abundance of *Faecalibacterium* species, as suggested by our data, are consistent with gut microbiome changes described in PLHIV in other studies (22, 23, 61). *Faecalibacterium prausnitzii*, a member of clostridial cluster IV, is one of a number of obligate anaerobic gut commensal bacteria that are responsible for the production of the short chain fatty acid butyrate through the fermentation of carbohydrates in the colon by inducing (62). Butyrate, in turn, contributes to gut barrier function by inducing tight junction assembly through an AMPK-dependent pathway (63). In contrast, increased prevalence of *Prevotella* species is associated with increased susceptibility to gut inflammation through indirect suppression of IL-18 production (64). However, while it has been suggested that an increased presence of potentially pathogenic bacteria in the gastrointestinal lumen may induce expression of pro-inflammatory cytokines, thereby increasing epithelial layer permeability (65, 66), these changes in microbiota composition were not reflected in the confocal imaging results.

There are a number of reasons why interpretation of the microbiome data is difficult. First, the cohort was small, and the study was cross-sectional, not permitting longitudinal microbiome follow-up. Three participants were treated for a sexually transmitted infection with antibiotics before enrolment. Finally, it has been recognised that ART has a variable impact on the composition of the microbiome, and this could not be assessed in this study (67, 68).

Plasma sCD14 concentration is a measure of monocyte activation in response to lipopolysaccharide from the cell walls of gram-negative bacteria (45, 69). We did not find, however, an increasing trend in levels of sCD14 in CHI patients. Recent studies have revealed differing findings on the effect of ART on levels of sCD14 (45, 70, 71). This variability may be due to a number of confounding factors, such as the duration of ART, although variability persists when individual patients are followed longitudinally (71); behavioural factors such as smoking may also be implicated (72, 73). The measured levels of sCD14 varied highly between previously published studies, varying from 0.7x10^6^pg/mL (74) to 4x10^6^pg/mL (45) in HIV-uninfected controls. Our study’s median of 1.8x10^6^ pg/mL for HIV-uninfected controls and 1.4x10^6^ pg/mL for PLHIV is consistent with results from other studies (45, 74). No increasing trend in levels of TNF-α, IL-6 or systemic activated HLA-DR+CD38+ CD8+ T-cells was found in the CHI patients.

Presentation of intraluminal antigens from the intestinal lumen is part of normal physiology in the regulation of immune responses and establishment of immunotolerance (75, 76), and it is possible for increased bacterial products to enter the portal and systemic circulation due to dysregulation of immune lymphocytes despite maintenance of epithelial barrier integrity. HIV-1 can infect hepatic Kupffer cells, which express CD14 and other receptors responding to LPS, altering measured levels of these markers (77). However, partial restoration of Kupffer cells has been found following initiation of ART (78).

The strength of this study was the detailed comparison with HIV-uninfected controls, including not only the confocal imaging, but also the detailed study of immune activation locally and systemically, as well as of the microbiome. This study was however limited by its cross-sectional design. Hence, we were unable to demonstrate whether the lack of changes in intestinal permeability was due to recovery following ART initiation. A more significant limitation of this study was the small sample size, which increases the possibility of type II errors. Finally, the small sample including only men makes generalisability difficult. As a pilot study, the main aim was to identify trends in measures of the gastrointestinal barrier that could contribute to microbial translocation and contribute exploratory data to support further larger studies of barrier dysfunction and microbial dysbiosis.

In conclusion, our results indicate that despite slightly impaired CD4+ T-cell recovery in the gastrointestinal tissue of CHI patients, no changes to physical epithelial integrity were found, nor significantly increased activation of CD8 T-cells within GALT. Furthermore, microbial translocation and inflammatory markers trended towards return to baseline. Our study raises doubts about the significance of microbial translocation and systemic inflammation in HIV-positive participants on effective ART. Analyses have suggested that there is little difference in life expectancy in PLHIV compared to HIV-uninfected individuals when controlling for other risk factors (79), although a more recent study suggests that PLHIV who commenced ART in the US between 2011-2016 with high CD4 counts still had about 7 years less life expectancy, plus more years of comorbidities. Several issues remain, such as whether dysregulation of intestinal CD4+ lymphocytes alone facilitates increased passage of bacterial by-products into the portal circulation. In summary, our study suggests that the importance of microbial translocation and its contribution to systemic immune activation in well-controlled HIV-positive patients may not be as significant as widely reported and believed.

## Data Availability Statement

The raw data supporting the conclusions of this article will be made available by the authors, without undue reservation.

## Ethics Statement

This study was approved by the St Vincent’s Human Research Ethics Committee (HREC 14/214). All participants provided written informed consent. The patients/participants provided their written informed consent to participate in this study.

## Author Contributions

Experimental work and data analysis: GM, JZ, MB, NS, GR, LL, and MD. Project conception: MD, MAB, KK, AK, and TP. Patient recruitment and procedures: MD, GM, KK, and AK. Manuscript written by GM, JZ, and MD. All authors contributed to the article and approved the submitted version.

## Funding

The study was funded by a St Vincent’s Clinic Foundation Project Grant. Cancer Institute NSW Equipment Grant 10REG114, Australia Research Council (ARC) Linkage Project LP10020080. TP is supported by National Health and Medical Research Council (NHMRC) Senior Research Fellowship (APP1155678) and the Ernest Heine Family Foundation. JZ was supported by NHMRC Fellowship 1063422 and ADK by NHMRC Program Grant 1052979.

## Conflict of Interest

The authors declare that the research was conducted in the absence of any commercial or financial relationships that could be construed as a potential conflict of interest.

The handling editor has declared a shared affiliation, though no other collaboration with one of the authors NS at the time of review.

## Supplementary Material

Supplementary Figure 1 – Representative flow plots and gating of CD8 T-cell subsets. **(A)**– Top row: Gating of CD45+ SSC^low^ lymphocytes, then CD3+ and EpCAM-negative, then CD45RA-negative, then CD8 T-cells (CD3+ CD4 negative). -Bottom row: CD8 T-cells then analysed for CD38 *vs* HLA-DR and CD38 *vs* CD127. -Then CD38+HLA-DR+ and CD38+CD127^low^ cells, respectively, were analysed for CD103 expression. **(B)**– Top row: Gating of CD45+ SSC^low^ lymphocytes, then CD3+ and CD20-negative, then CD45RA-negative, then CD8 T-cells (CD3+ CD4 negative). -Bottom row: CD8 T cells then analysed for CD38 *vs* HLA-DR, and CD49d *vs* integrin ß7, and CD38+CD127^low^ cells. -Then CD38+HLA-DR+ cells were analysed for CD49d *vs* integrin ß7 expression

Supplementary Figure 2 Activated CD38+CD127^low^ CD8 T-cells in blood and tissues. Activated CD38+CD127^low^ as % of CD45RA- CD8 T cells in peripheral blood, and in TI and LC biopsies, by study group. HUC: HIV-uninfected controls, PHI: HIV-positive patients treated during primary infection, CHI: HIV-positive patients treated during chronic infection.
