# Supplementary figures and images for: Preservation of Gastrointestinal Mucosal Barrier Function and Microbiome in Patients With Controlled HIV Infection
Source: Front Immunol. 2021 May 31;12:688886. doi: 10.3389/fimmu.2021.688886 (PMC8203413; doi:10.3389/fimmu.2021.688886)

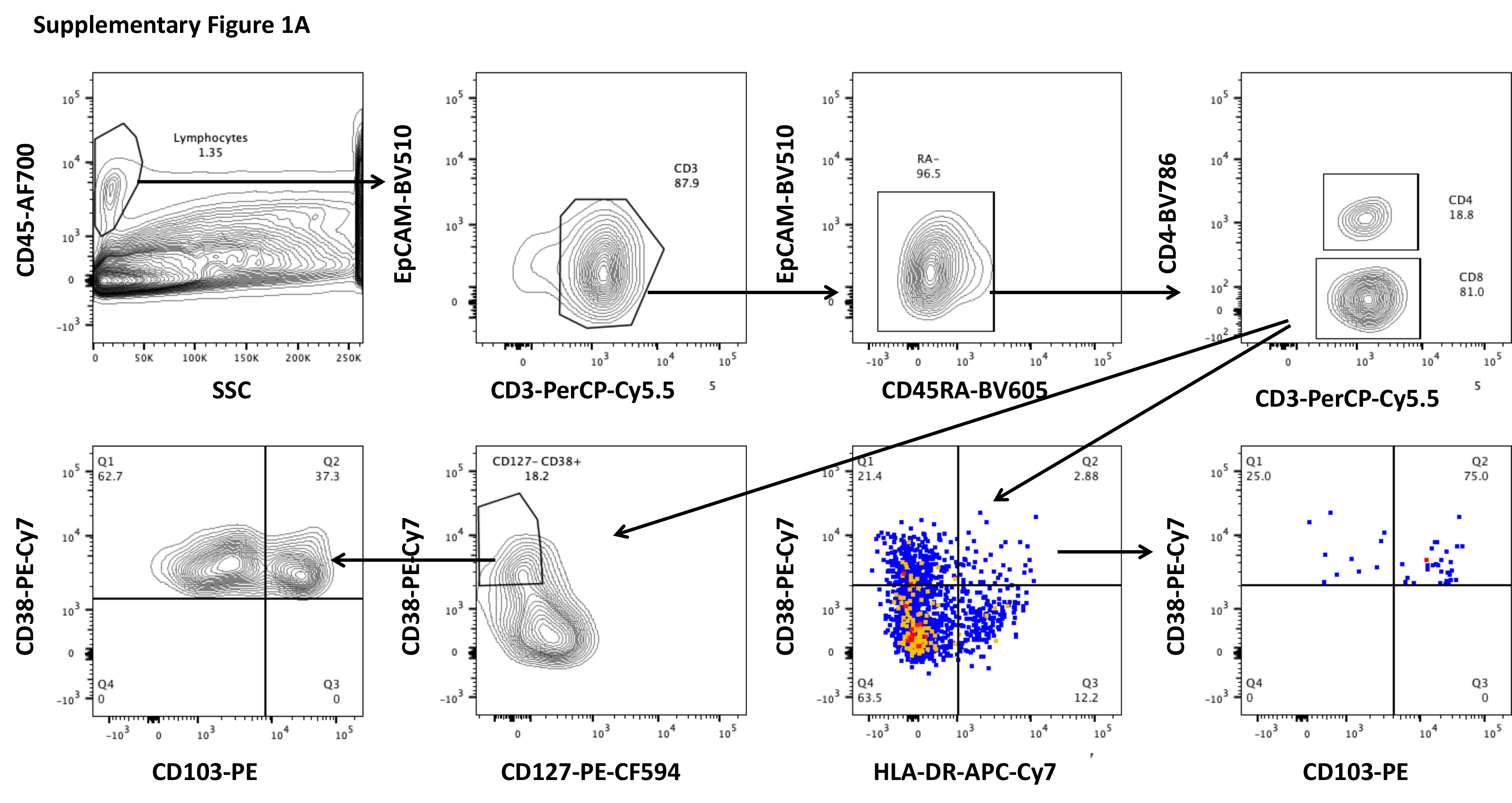

Supplement: Supplementary Figure 1 — – Representative flow plots and gating of CD8 T-cell subsets. (A)– Top row: Gating of CD45+ SSClow lymphocytes, then CD3+ and EpCAM-negative, then CD45RA-negative, then CD8 T-cells (CD3+ CD4 negative). -Bottom row: CD8 T-cells then analysed for CD38 vs HLA-DR and CD38 vs CD127. -Then CD38+HLA-DR+ and CD38+CD127low cells, respectively, were analysed for CD103 expression. (B)– Top row: Gating of CD45+ SSClow lymphocytes, then CD3+ and CD20-negative, then CD45RA-negative, then CD8 T-cells (CD3+ CD4 negative). -Bottom row: CD8 T cells then analysed for CD38 vs HLA-DR, and CD49d vs integrin ß7, and CD38+CD127low cells. -Then CD38+HLA-DR+ cells were analysed for CD49d vs integrin ß7 expression [file Image_1.tif]

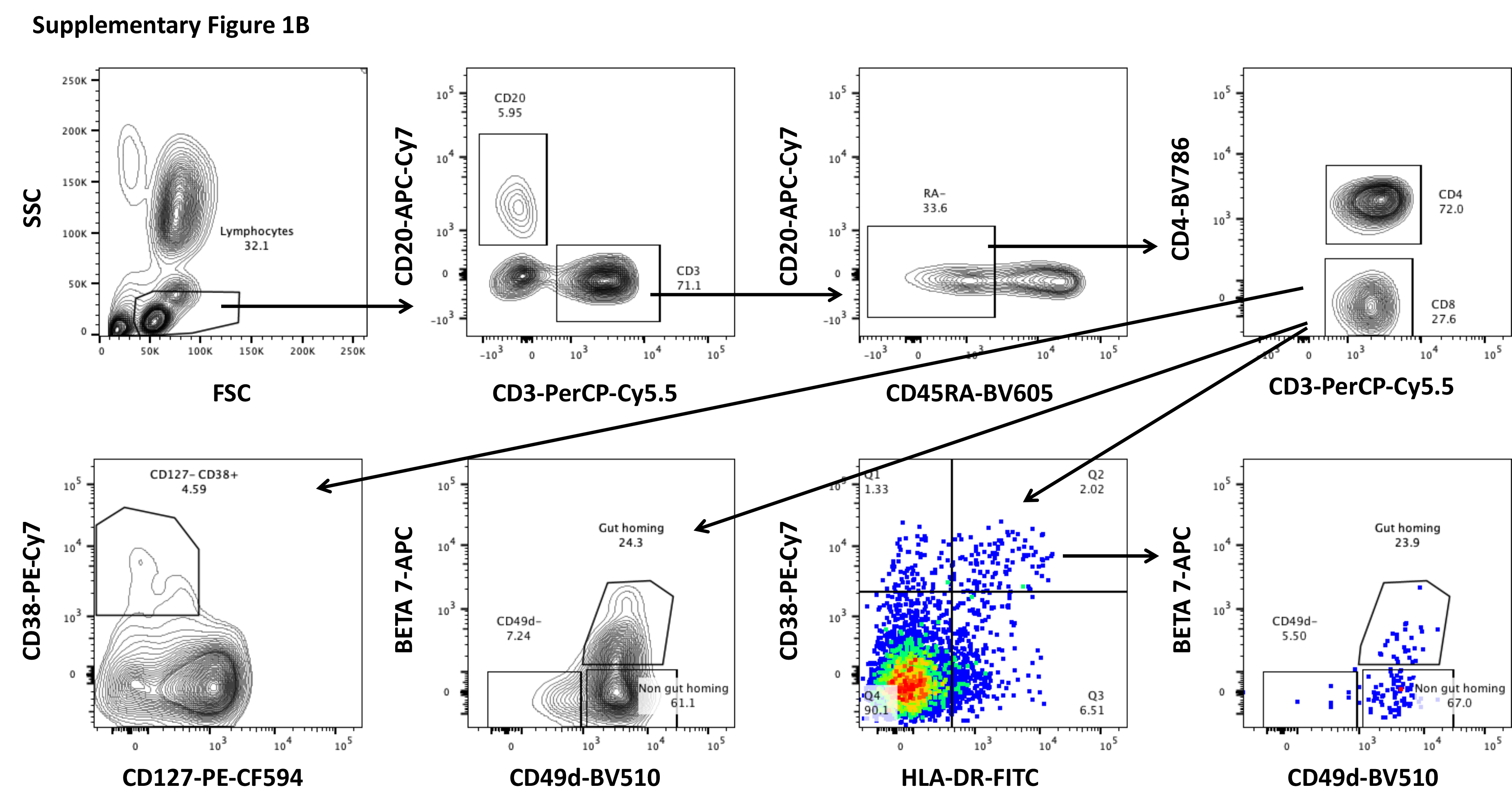

Supplement: Supplementary Figure 2 — Activated CD38+CD127low CD8 T-cells in blood and tissues. Activated CD38+CD127low as % of CD45RA- CD8 T cells in peripheral blood, and in TI and LC biopsies, by study group. HUC: HIV-uninfected controls, PHI: HIV-positive patients treated during primary infection, CHI: HIV-positive patients treated during chronic infection. [file Image_2.tif]
